# Signal detection models as contextual bandits

**DOI:** 10.1098/rsos.230157

**Published:** 2023-06-21

**Authors:** Thomas N. Sherratt, Erica O'Neill

**Affiliations:** Department of Biology, Carleton University, 1125 Colonel By Drive, Ottawa, Ontario, Canada K1S 5B6

**Keywords:** decision theory, signal detection theory, multi-armed bandit, contextual bandit, Softmax, Thompson sampling

## Abstract

Signal detection theory (SDT) has been widely applied to identify the optimal discriminative decisions of receivers under uncertainty. However, the approach assumes that decision-makers immediately adopt the appropriate acceptance threshold, even though the optimal response must often be learned. Here we recast the classical normal–normal (and power-law) signal detection model as a contextual multi-armed bandit (CMAB). Thus, rather than starting with complete information, decision-makers must infer how the magnitude of a continuous cue is related to the probability that a signaller is desirable, while simultaneously seeking to exploit the information they acquire. We explain how various CMAB heuristics resolve the trade-off between better estimating the underlying relationship and exploiting it. Next, we determined how naive human volunteers resolve signal detection problems with a continuous cue. As anticipated, a model of choice (accept/reject) that assumed volunteers immediately adopted the SDT-predicted acceptance threshold did not predict volunteer behaviour well. The Softmax rule for solving CMABs, with choices based on a logistic function of the expected payoffs, best explained the decisions of our volunteers but a simple midpoint algorithm also predicted decisions well under some conditions. CMABs offer principled parametric solutions to solving many classical SDT problems when decision-makers start with incomplete information.

## 1. Introduction

Signal detection theory (SDT) is a general Bayesian framework used to identify optimal discriminative decisions under uncertainty [1,2]. At its simplest, an observer (the ‘receiver') evaluates the probability of a stimulus (e.g. a rustle of leaves) being from a ‘signaller' of type A (e.g. the wind) or type B (e.g. a predator) and uses this information to determine its response. If the receiver's best response is dependent on the true nature of the signaller (e.g. it should continue to forage if the signaller is type A and run away if type B) and the two types of signaller are only partially discriminable, then the payoff-maximizing decision will depend on not just the stimulus, but also the underlying (base) probability of the signaller being type A and the payoffs from correct (A treated as A, B as B) and incorrect (A treated as B, B as A) decisions.

The most widely invoked SDT models assume that the two types of signaller overlap in their perceived appearance in a single stimulus dimension, with the degree of overlap inversely related to their discriminability. For example, in the popular normal–normal equal variance SDT model, the probability distributions of individual observations *x* from type A and type B signallers are assumed to be normal with means of *μ_A_* and *μ_B_*, respectively, each with variance *σ*^2^ [3,4]. Likewise, the ‘power law' SDT model [5,6] assumes that the continuous probability distributions of perceived appearances of the two types of signaller are each negative exponential distributions with means *μ_A_* and *μ_B._* In both cases, when *μ_A_* < *μ_B_* then the likelihood of a signaller being type B increases monotonically as the size of the stimulus *x* increases, so that receivers should treat a signaller as if it were type A if its perceived appearance *x* is below a threshold (x*), and otherwise treat the signaller as type B. SDT models identify this perceived appearance threshold (or thresholds) and elucidate its implications, sometimes with surprising results [4,7,8].

SDT has found wide application in a range of disciplines from psychology to evolutionary biology [8–10]. However, despite its intuitive appeal, SDT models are limited in one important way: they pre-suppose that the receiver behaves as if it knows all the relevant model parameters at the outset [11]. Indeed, to fit this assumption of ‘complete information', standard tests of SDT often pre-expose receivers to signallers before formal data collection begins [12–15] and may even reject subjects that fail to meet a training threshold [16]. In natural systems, the responses of receivers towards unfamiliar signallers may be subject to selection, particularly if these decisions have important fitness consequences. So, after a long and stable evolutionary history, receivers may be selected to respond appropriately to unfamiliar signallers, as if they had complete information. However, a more flexible strategy based on learning will be needed if receivers live in spatially heterogeneous or temporally variable signalling environments [6,17,18] or under conditions in which stimuli are so diverse that responses of receivers cannot be ‘hard-wired'. Indeed, the fact that chemically defended species with conspicuous warning signals often evolve a similar appearance to share the cost of educating predators (a phenomenon known as ‘Müllerian mimicry') strongly suggests that avoidance responses frequently have to be learned [19].

How can receivers make strategic discriminative decisions if they start out knowing little? Imagine an environment containing desirable type A signallers, which yield benefit *b* if accepted, and undesirable type B signallers, which incur cost *c* if accepted. Acceptance therefore gives an uncertain reward. By contrast, we let rejection yield a certain reward of 0. Let us further assume that the two types of signaller can be probabilistically discriminated on the basis of a trait *x*. Unlike a classical SDT problem, we assume that the receiver does not adopt the optimal acceptance threshold from the outset. Instead, it has to estimate the relationship between *x* and the likelihood that the signaller is desirable while simultaneously seeking to maximize its long-term payoff. If a receiver were to simply accept all signallers, then it would learn the most about the relationship between *x* and the probability that a signaller is desirable, but it would not have used this information to reject signallers it believes are undesirable. Conversely, if the receiver seeks only to maximize its immediate gain, then it may reject many desirable signallers because of its limited understanding. Exploration–exploitation models (also called ‘multi-armed bandit models' after a standard exemplar) identify ways in which receivers can optimally balance information gathering (exploration) and information use (exploitation) [20]. Moreover, in problems where a cue (side information) is available to help indicate the profitability of choosing a particular option (such as accept), then the model is said to be a ‘contextual multi-armed bandit' (CMAB) [21,22].

The first goal of our paper is to highlight the fundamental relationship between SDT and CMAB models. This close relationship arises from their common assumptions: both SDT and CMAB models seek to identify payoff-maximizing choices under uncertainty, and both are framed in Bayesian terms. However, SDT models effectively assume highly informative priors that reflect the true nature of the signalling environment, whereas CMABs can reflect any level of prior beliefs, including completely uninformative priors.

Naturally, to predict the probability that a newly encountered signaller with appearance *x* is type A, an uninformed receiver needs to start with an appropriate general model of the relationship between *x* and the probability of being type A. The receiver can then parametrize this model based on what it has learned. Appendix 1 in our electronic supplementary material demonstrates that both the classical normal–normal equal variance and power-law SDT models dictate that the log odds of a signaller being type A is linearly related to its appearance *x* (see also [23]). So, the (posterior) probability distribution that any newly encountered signaller with appearance *x* is type A is most appropriately inferred from the fit of a binary logistic model to the characteristics (appearance and signaller type) of those signallers that have been accepted so far, combined with a prior. Clearly, a naive decision-maker will not know precisely how a signaller's appearance *x* is quantitatively related to the probability that it is a given type, so to argue that decision-makers start out parametrizing a logistic model may seem a bold assumption. However, the use of a logistic model to represent the odds ratio of a binary outcome with a continuous predictor is quantitatively reasonable from a statistical perspective, since it is the maximum entropy distribution (i.e. the least informative model consistent with there being a relationship) [24]. Of course, a receiver's impression of a sigmoidal relationship will be further reinforced as it discovers that signallers with extreme values of *x* are predominantly one type or another, while signallers with intermediate *x* are more likely to be a mix of the two types.

Even if the underlying model that needs to be parametrized can be assumed, identifying optimal sampling rules (e.g. when to accept a signaller) for even simple bandit problems is computationally demanding (e.g. via the Gittins index [25] or dynamic programming [26]). Given this complexity, it is clear that most decision-makers will only be able to find an approximate solution to exploration–exploitation problems through the application of an efficient heuristic [27,28]. Several such heuristics have been proposed, most of them Bayesian in nature (see Methods). The relative efficiency of different heuristics in resolving multi-armed bandits, including contextual bandits with continuous cues, has been widely discussed [21,29,30]. Therefore, rather than present a detailed comparison of the efficiency of the heuristics here we simply confirm that so long as the CMAB heuristics are sufficiently exploratory, they can eventually accurately parametrize the underlying logistic model.

Having argued that signal detection models can be represented as CMABs and established that several of the heuristics come closer to gaining complete information as time continues, the next question is which CMAB heuristic best explains how receivers resolve this type of exploration–exploitation problem in practice. To address this question, we conducted an online experiment in which human volunteers were presented with desirable signallers (which yield a fixed benefit on acceptance) and undesirable signallers (which incur a fixed cost on acceptance) that could only be probabilistically discriminated based on the magnitude of a continuous cue (in this case their greyness). In contrast to most of the previous work seeking to test predictions of SDT, our volunteers were completely naive, and we were interested not simply in whether they were able to solve the problem in an efficient way but how they solve it.

## 2. Methods

### 2.1. The general problem

Let a receiver sequentially encounter a series of individual signallers, each time deciding whether to accept or reject the signaller based on its continuous trait (*x*). The receiver gains a known benefit *b* if it accepts a signaller of type A (a desirable signaller) but pays a known cost *c* if it accepts a signaller of type B (an undesirable signaller). In this way, acceptance of a signaller provides an uncertain immediate payoff (a cost or benefit) but also more information (which the receiver can use in the future) about the relationship between a signaller's appearance and its probability of being desirable. By contrast, we assume rejection generates a certain payoff of 0 but provides no information. Since only one of the two ‘arms' has an uncertain reward, and there is ‘side information' indicating the desirability of the signaller, this model is in effect a ‘one-armed’ contextual bandit [31].

In contrast to SDT models, the receiver does not know the optimal SDT-predicted appearance threshold for acceptance (x*; see electronic supplementary material, appendix 2) at the outset. Instead, by basing its inferences on the assumption of a linear relationship between the continuous trait *x* and a signaller's log odds ratio of being type A (see electronic supplementary material, appendix 1), the receiver can use the characteristics of signallers it has so far accepted (if any) to determine the posterior probability distribution (*p*) that the newly encountered signaller with given appearance is type A. This posterior distribution provides the basis for decision-making by most of the heuristics we now consider, although none of these heuristics use the optimal acceptance threshold x* directly.

### 2.2. Heuristic solutions to the exploration–exploitation problem

A receiver adopting a Thompson sampling (TS) heuristic [32] would select one value (= *p_i_*) at random from its current posterior belief distribution *p* that the signaller is type A. If *p_i_b* − (1 − *p_i_*)*c* > 0 then it should accept the signaller since its sampled payoff is higher than that of the alternative arm (reject) with a payoff of 0. Accordingly, the mean probability of the Thompson sampler accepting a signaller with appearance *x* is 1 minus the cumulative probability that the payoff is less than 0.

Most other decision heuristics use point estimates of the current posterior belief distribution *p*, notably the expectation. In particular, the Greedy algorithm [33] looks only to exploit its current information and will accept a signaller if the expected payoff from accepting the signaller $\left(v_{\mathrm{accept}}\right)$ exceeds that of rejection $\left(v_{\mathrm{reject}}\right)$, i.e. $\;\bar{\;p}\;b-(1-\bar{\;p})c\;$ greater than 0 where $\bar{\;p}$ is the mean posterior probability that the signaller with appearance *x* is type A. In seeking only to exploit its current information, Greedy can sometimes fail to sufficiently explore. To introduce an arbitrary amount of exploration through ‘dithering', ε-Greedy adopts Greedy with probability 1 − ε, the alternative strategy being that it accepts signallers at random with a probability of 0.5 [30].

While Greedy is an all-or-nothing heuristic (a step function), the Softmax (or Boltzmann [34]) rule assumes a smoother sigmoidal relationship, with the probability of accepting a signaller assumed to be an S-shaped function of the expected payoffs from accepting compared to rejecting it, namely $\exp(\;v_{\mathrm{accept}\;}/\tau )/(\exp(\;v_{\mathrm{reject}\;}/\tau )\;\;+\exp(v_{\mathrm{accept}\;}/\tau ))$. Here, $\exp(v_{\mathrm{reject}\;}/\tau )=1$ since $v_{\mathrm{reject}\;}=0$, while *τ* is simply a ‘temperature' parameter that affects the slope of the relationship. Note that the Softmax decision rule becomes the Greedy rule (probability of acceptance 0 or 1) as *τ* approaches 0 and becomes a Random rule with a limiting probability of acceptance of 0.5 as *τ* approaches infinity. Other heuristics encourage exploration by being more optimistic about the payoff from choosing arms with uncertain rewards. In particular, the upper confidence bound (UCB) [35] heuristic adopts the highest plausible estimate of the profitability of accepting a signaller, accepting if *p_u_b* − (1 − *p_u_*)*c* > 0, where *p_u_* is the upper value of highest probability density interval (HPDI) which encompasses a proportion (1 − *γ*) of *p* (e.g. *γ* = 0.05).

### 2.3. Demonstration that the different heuristics can learn the underlying relationship

All numerical simulations were conducted in R [36]. To demonstrate how two of the best-known CMAB heuristics, TS [33] and Softmax [34], are able to parametrize the underlying logistic model, we ran simulations in which we presented naive receivers with a sequence of signallers. These signallers were type A (desirable) with given probability *ρ* (the base rate), the alternative being that they were type B. The appearance of each signaller was characterized by a continuous trait drawn from a normal distribution with fixed variance, and population mean dependent on the signaller's type. At any point in time, receivers were able to estimate, through the fit of a logistic model to their history of accepted signallers, the probability distribution that a newly encountered signaller with appearance *x* is type A and act according to its strategy. Since the logistic model had to be repeatedly fitted in these simulations as more signallers were accepted, the estimates were based on a quadratic (normal) approximation of the posterior parameters, derived via the *quap* function of the *rethinking* package [37]. For flexibility, we assumed relatively wide normal priors in the fitted logistic model (logit intercept∼*N*(0, 10), logit slope∼*N*(0, 10); starting values for the intercept and slope were both 0).

### 2.4. How humans solve contextual bandits with continuous cues

While it is helpful to document the behaviour of different heuristics as they parametrize the underlying logistic model, it is also of interest to elucidate how organisms (in this case humans) solve these problems in practice. We therefore conducted an experiment. The experiment was administered through a Web application programmed in R (version 3.6.2 [36]) using the RShiny package [38]. Our volunteers (36 in total) were drawn primarily from biology undergraduate and graduate programmes at Carleton University, Ottawa with recruitment by email. Participants accessed the Web application via a URL link in the email invitation and were only engaged once. Before starting the experiment, participants were instructed to watch a short introductory video describing the procedure. No details of the experimental aims were given at that time, and no information was given concerning a potential relationship between a signaller's appearance and its true nature.

Our volunteers were presented with a series of computer-generated signallers (solid-coloured circles; see electronic supplementary material, figures S1 and S2) over a sequence of trials. In any given trial, the signaller was either desirable (good) or undesirable (bad) and signaller type could be probabilistically inferred from their appearance (their greyness, see below). Participants were told the benefits of a correct acceptance of a desirable signaller (1), the cost of an incorrect acceptance of an undesirable signaller (−1), and the number of trials they were to complete (50). They were also made aware they would not gain or lose any points for rejecting a signaller. With this limited information, participants were asked to accept or reject signallers with the aim of maximizing their total points by the end of the 50 trials. There was no time limit. To help motivate participants to maximize their returns, we highlighted the participant's cumulative points, provided immediate feedback from a range of phrases upon accepting a signaller (e.g. ‘Fantastic! + 1 point', ‘Oh no! −1 point') and displayed a points tier (‘Bronze rank: 5–8 points', etc.). Volunteers that accepted a signaller found out whether it was desirable or undesirable from their change in payoff and the above feedback. However, volunteers that rejected a signaller received no information, creating an exploration–exploitation dilemma.

Signallers were solid-coloured circles that varied only in their shade of grey. Setting R, G and B values identical (= C) produced a shade of grey between black (C = 0) and white (C = 255). When generating signallers, their greyness (i.e. value of C) was drawn randomly from one of two normal distributions with population means dependent on the nature of the signaller (µ_good_ and µ_bad_). As with the classical normal–normal SDT model, the variance in C was the same for the two types of signaller. There were four treatment groups in a factorial design with 2 levels of discriminability (based on differences in the population mean greyness of the two signaller types) and 2 levels of base rate. In high discriminability treatments, µ_good_ and µ_bad_ were 90 and 165, respectively (with a common standard deviation of 25, this represents a difference of 3 s.d. units). In low discriminability treatments, µ_good_ and µ_bad_ were 115 and 140, respectively (a difference of 1 s.d. unit). In all cases, the vast majority of sample values of C fell between 0 and 255, although any draw of C outside this range was truncated. The underlying probability of a signaller being good in any given trial (base rate) was either high (0.7) or low (0.3). Subjects were invited to play one of the four treatments according to their birth month (January–March; April–June; etc.). Consequently, our sample sizes for the different versions were similar but not identical (*n* = 8 or 10). For each trial, we recorded whether the signaller was good or bad, the C value of the signaller, the choice made by the participant (accept or reject), and the participant's cumulative points based on its acceptances of good and bad signallers.

### 2.5. Statistical analysis of human choice data

Our primary aim was to identify which heuristic exploration–exploitation model, from a range of candidate alternatives, best predicted human choice (accept/reject) behaviour. To do so, we used Stan (https://mc-stan.org/) to fit and compare multi-level models of human choices. Stan was accessed in R [36] via RStan and the models were coded using the *ulam* function in the *rethinking* package [37]. All posterior distributions were estimated using Markov chain Monte Carlo sampling for 4000 iterations in four separate chains. To facilitate model fitting, the RGB values (C) of all signallers were rescaled by dividing C by 255, ensuring a value of perceived appearance (*x*) between 0 and 1.

In addition to the heuristic exploration–exploitation models described previously, we also considered the plausibility of two simple non-Bayesian strategies that are independent of the associated payoffs. The first is an entirely random strategy in which receivers accept signallers, whatever their value of *x*, with a probability of 0.5. The second heuristic involved receivers simply adopting the midpoint between the sample mean appearance of desirable signallers so far accepted $\left(\bar{x_{d}}\right)$ and the sample mean appearance of undesirable signallers so far accepted $\left(\bar{x_{u}}\right)$ as the threshold below or above which (depending on which sample mean is greater) signallers should be accepted. The Midpoint strategy accepted all signallers until at least one desirable and at least one undesirable signaller had been accepted, allowing the sample mean appearances of the two signaller types to be estimated (continually updated as new signallers are accepted).

To identify the most plausible behavioural rule guiding decision-makers, we first identified the probability (*p*_Strategy_, where Strategy ∈ TS, Softmax, Greedy, UCB, Random or Midpoint) of a given heuristic accepting a newly encountered signaller with appearance *x* based on the information that had been accumulated so far by the volunteer (the appearance of each previously accepted signaller, and whether they were good or bad). For each of the 1800 (36 × 50) signallers presented to volunteers, we could therefore predict how an individual receiver following these heuristics should act when it encounters a signaller with a certain appearance given its history of accepted signallers and compare these predictions with how the volunteers actually responded. By basing the heuristic response on the actual encounter and decision history of our volunteers prior to encountering a given signaller (i.e. the appearances of signallers so far accepted, along with their type) the model was ‘yoked' directly to our volunteer experiences [39].

No model will provide a perfect representation of the behaviour of our volunteers. To introduce a ‘trembling hand' to our models, in which individuals do not exclusively use a given heuristic, we allowed individuals to deviate from the given heuristic with a probability *ε* (analogous to ε-Greedy). Thus, with probability (1 − *ε*) the volunteer followed the heuristic, but with probability *ε* we assumed that volunteer *i* would accept a signaller with an individual-specific probability *q*_i_. In this way, while individuals were assumed to deviate from the heuristics at the same rate, the probability of the receiver accepting the signaller when it deviated was allowed to vary from individual to individual. The probability of acceptance of a given signaller was therefore given by

$$\varepsilon q_{i}+(1-\varepsilon )p_{\mathrm{Strategy}}.$$

To compare the out-of-sample predictive accuracy of our models, we used Pareto-smoothed importance sampling cross-validation (PSIS) [40], computed point by point using a function in the *rethinking* package. We allowed *ε* to be estimated by treating it as a free parameter (its prior was assumed to be uniform, distributed between 0 and 1). The prior for the logit of *q*_i_ was assumed to follow a normal distribution with mean 0 (hence a mean of 0.5 for *q*_i_) and an exponentially distributed standard deviation with rate parameter 1. We again assumed that volunteers using the above heuristics began with relatively uninformative normal priors for the logistic model (intercept∼*N*(0, 10), slope∼*N*(0, 10); starting values for intercept and slope were 0). Since parameter *τ* of the Softmax model is constrained to be positive and likely to be relatively low, we set its prior to be exponential with rate parameter 1. We assumed the upper probability for the UCB heuristic was the upper bound of the 0.95 HPDI, i.e. *γ* = 0.05.

We began by assuming that our volunteers used the same rules to guide their decision-making across all four treatments, fitting the models to the combined data. However, it is possible that our volunteers adopted different strategies under different conditions. To account for this possibility, we also fitted the same models separately to data from each of the four treatments. Despite the lack of nesting, we refer to such models as ‘multi-level' since individual acceptance probability had a prior with a (hyper)parameter that itself followed a distribution [37]. To validate our modelling approach, we repeatedly simulated the responses of eight virtual volunteers (the minimum sample size per treatment) behaving according to one of the seven heuristics (Softmax, TS, Greedy, Midpoint, UCB, SDT, Random) over 50 encounters with signallers, with each virtual volunteer having a fixed rate of choosing a response at random (see electronic supplementary material for details). By fitting the multi-level models to these simulated data and comparing out-of-sample predictive accuracy using PSIS to select the most probable generative model, we confirmed that this approach was able to correctly identify the data-generating model (for example, in 10 out of 10 simulations it correctly identified Softmax as the generating heuristic, and in 10 out of 10 simulations it correctly identified TS as the generating heuristic, both in treatment 1 (low discriminability, low base rate) and treatment 4 (high discriminability, high base rate)). This approach was also able to derive reasonable estimates of the underlying parameters (see electronic supplementary material).

## 3. Results

### 3.1. Heuristic solutions to contextual bandits with continuous cues

Any exploration–exploitation heuristic that involves (i) Bayesian learning and (ii) a continued incentive to accept at least some signallers, will gain increasingly accurate and precise estimates of the true relationship between the trait *x* of a signaller and its probability of being desirable.

The TS, UCB, Softmax and ε-Greedy heuristics all have these properties. For example, electronic supplementary material, figure S3, shows that both the TS and Softmax strategies come closer to estimating the true underlying model as more information is gained from accepting signallers. Moreover, since the TS and UCB heuristics incorporate the information they gain in a simple payoff-dependent acceptance rule akin to that used by SDT, then they will necessarily converge on a mean payoff equivalent to that obtained using the optimal SDT-predicted acceptance threshold (x*). While the Softmax heuristic ultimately gains accurate estimates of the probability that a given signaller is desirable, rather than using a hard acceptance threshold (with a probability of acceptance either 1 or 0), the acceptance rate of the heuristic is a smoother sigmoidal function of *x* with the point of inflection around the value of *x* at which the signaller is equally likely to be desirable and undesirable; so its long-term average payoff will be lower. The ε-Greedy heuristic likewise gains accurate estimates, but since it follows a random sampling strategy with probability *ε* it will never achieve the payoff of the optimal solution.

**Figure 1.**
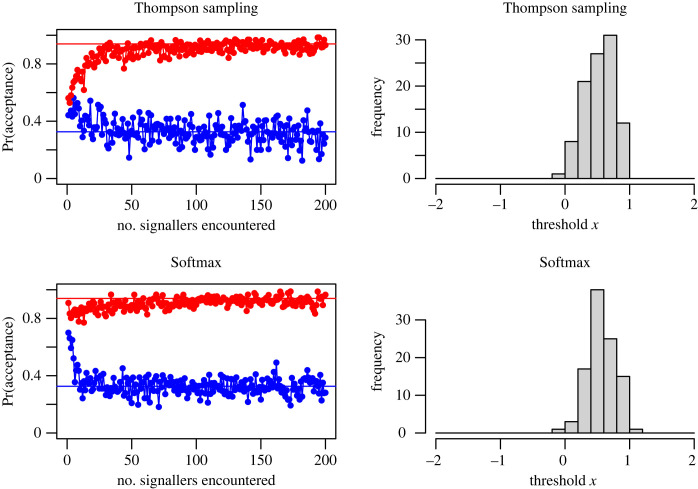
The average probabilities (calculated over 100 separate simulations) of acceptance of a desirable signaller of type A (red) and undesirable signaller of type B (blue) by a receiver that employs (first row) TS and (second row) Softmax (with *τ* = 0.1) as an exploration–exploitation heuristic over 200 encounters. Here type A and type B have normally distributed appearances with means *μ_A_* and *μ_B_* respectively, and standard deviation *σ*. Model parameters: *b* = 2, *c* = 1, *μ_A_* = −1, *μ_B_* = 1, *σ* = 1, *ρ* = 0.6. The continuous red and blue lines represent the proportions of each signaller type A and B that should be accepted on encounter, given the SDT-predicted optimal threshold. Over time the receivers learn the relationship between a signaller's appearance and its log odds of being desirable and ultimately accept approximately the SDT-predicted proportions of signallers. The histograms show the distributions of the thresholds below which it is profitable to accept a signaller after 200 encounters (although neither of the heuristics use this threshold directly, TS will ultimately behave as if it does). See electronic supplementary material, figure S3, for the full posterior estimates of the logistic distribution after a given number of encounters.

To see how TS and Softmax strategies are able to converge on an appearance-based acceptance criterion close to the optimal SDT threshold, the first row of figure 1 shows the mean (over 100 replicate simulations) proportions of type A and type B signallers accepted over time by a Thompson sampler encountering a randomly generated sequence of signallers. Given the high initial uncertainty and the fact that the TS heuristic samples from the full posterior distribution, the initial proportion of each signaller type accepted on encounter was intermediate. However, as more experience is gained, the decision to accept a signaller becomes increasingly sensitive to *x*. Ultimately, the acceptance rate of each signaller type comes close to the SDT-predicted proportions of type A and type B signallers that should be accepted on encounter based on the optimal appearance threshold (x*) for acceptance (electronic supplementary material, appendix S2). The second row of figure 1 shows analogous results for the Softmax heuristic. The high initial acceptance rate of Softmax arises because the heuristic uses expectations rather than the full distributions and with *b* > *c* in this demonstration the expected probability that a newly encountered signaller will be profitable to accept will be high.

Under the conditions assumed in figure 1, the SDT-predicted threshold below which signallers should be accepted is 0.549. The second column of figure 1 shows the distribution of these thresholds under the TS and Softmax heuristics after 200 encounters. While there is some variation, the mean appearance threshold below which the expected payoff would exceed 0 (hence be more likely to accept than reject) that was eventually learned by Thompson samplers and Softmax heuristic after 200 encounters was 0.536 (s.d. 0.221) and 0.550 (s.d. 0.223), respectively.

### 3.2. How humans solve one-armed contextual bandits with continuous cues

**Figure 2.**
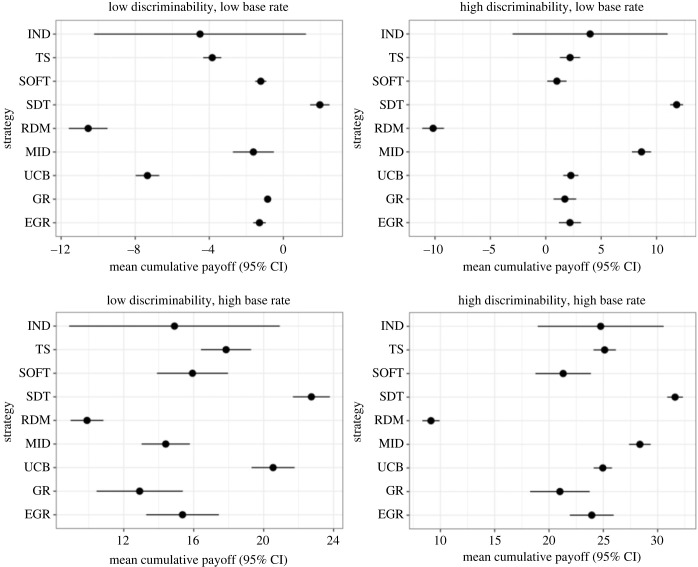
The mean cumulative payoff (with 95% CI) gained by volunteers (IND) in the experiments (four treatments comprising two levels of discriminability and two base rates) after 50 encounters with signallers. For comparative purposes, we show the mean cumulative payoff (with 95% CI) after 50 encounters with signallers for a range of exploration–exploitation heuristics, calculated over 100 separate simulations under the same mean conditions. TS, Thompson sampling; SOFT, Softmax (*τ* = 0.1); SDT, signal detection theory threshold; RDM, accept with 50% probability; MID, Midpoint; UCB, upper confidence bound (*γ* = 0.05); GR, Greedy; EGR, ε-Greedy (*ε* = 0.05).

Figure 2 illustrates the performance of our human volunteers by showing their mean cumulative payoff (with 95% CI) after 50 encounters with signallers under the four treatment conditions (for individual variation in payoffs, see electronic supplementary material, figure S4). For reference, we also include the mean cumulative payoff (with 95% CI) after 50 encounters with signallers of the different exploration–exploitation rules (necessarily with specific values of parameters), calculated over 100 separate simulations under the same mean conditions. In general, the mean cumulative payoff gained by human volunteers after encountering 50 signallers was significantly less than the mean cumulative SDT payoff would have been (paired *t*-tests, all *p* < 0.01, electronic supplementary material, table S3) and significantly higher than that gained by random chance with 50% acceptance rate (paired *t*-tests, all *p* < 0.021, electronic supplementary material, table S3) with an identical sequence of signallers. Our volunteers also tended to perform better than random if the random chance acceptance rate reflected the underlying base rate (electronic supplementary material, table S3).

While humans gained more than a random acceptance strategy and less than the SDT strategy with complete information, the same cumulative payoffs could conceivably be obtained in a range of different ways, so they offer only a crude insight into the actual strategies employed. To identify the exploration–exploitation rule that is most consistent overall with the way humans behaved, we compared the out-of-sample deviance of the fit of a range of choice models using PSIS. The Softmax strategy had the overall lowest out-of-sample deviance (table 1). Overall, the mean posterior estimates of the best fitting (Softmax) model were 0.29 (s.e. 0.03) for *ε* and 0.27 (s.e. 0.03) for the temperature parameter *τ* (see electronic supplementary material, figure S5, for the full posterior distributions). As expected, the omniscient SDT model and the Random model had relatively high out-of-sample deviance indicating a poor fit.

**Table 1.** Comparison of the fit of a range of exploration–exploitation models to our human choice data (all treatments combined) using PSIS. Models with low PSIS have the lowest estimated out-of-sample deviance (highest predictability). See electronic supplementary material for separate model fits to each of the four treatments.

	PSIS	SE	dPSIS	dSE	pPSIS	weight
Softmax	1844.92	40.17	0	n.a.	21.94	1
Thompson	1863.72	40.52	18.8	8.7	25.96	0
Midpoint	1865.88	42.53	20.96	31.79	30.81	0
SDT	1901.97	42.01	57.05	28.32	30.26	0
Greedy	1931.36	41.42	86.44	18.98	29.32	0
UCB	2181.43	32.39	336.5	32.95	30.74	0
Random	2231.42	30.48	386.5	34.49	31.54	0

The Softmax strategy continued to be one of the best predictors of volunteer choices when the models were fitted separately under the four different treatments. However, here the Midpoint strategy best explained human responses, except when the base rate was high and the discriminability of signallers was low. Under this condition, the Midpoint rule explained human data poorly (electronic supplementary material, tables S4–S7). Perhaps one reason why human behaviour is not well explained by the Midpoint strategy under this specific condition is the strategy's relatively low payoff. The derivation of the optimal thresholds in appendix S2 of the electronic supplementary material shows that the optimal acceptance threshold is the midpoint, i.e. $(\mu_{A}+\mu_{B})/2$, plus $\left(\sigma^{2}\mathrm{Log}(c(1-\rho )/b\rho ))/(\mu_{A}-\mu_{B}\right)$ which can be positive or negative, dependent on model parameters. So, the less discriminable the signallers (i.e. the lower $\left|\left(\mu_{A}-\mu_{B}\right)\right|$) the further the optimal acceptance threshold will be from the midpoint between the two means (see Discussion).

**Figure 3.**
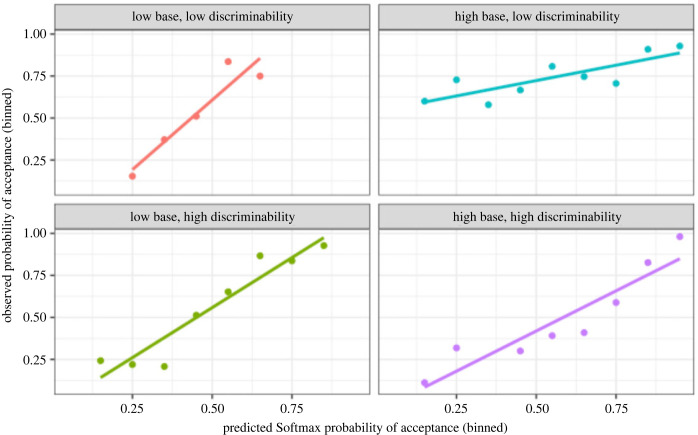
The predicted mean probability of accepting a signaller under the Softmax heuristic (binned in 0.1 intervals; 0–0.099, 0.1–0.199, …, 0.9–1) against the observed proportion of signallers accepted by volunteers for this prediction interval. The fitted lines are linear regressions. The Softmax predictions were obtained by fitting the Softmax model to the history of signallers accepted in each of the four treatments (assuming *ε* = 0). The posterior means of the temperature parameter *τ* under these conditions were as follows: low base rate, low discriminability, *τ* = 0.9 (s.e. 0.22); high base rate, low discriminability, *τ* = 0.38 (s.e. 0.04); low base rate, high discriminability, *τ* = 0.48 (s.e. 0.04); high base rate, high discriminability, *τ* = 0.37 (s.e. 0.03).

Naturally, even if the Softmax model has the (overall) highest out-of-sample predictability, a better fit does not necessarily mean a good fit. However, figure 3 shows that the Softmax strategy explained the volunteer choice behaviour relatively well, in that across all four treatments our volunteers rarely accepted signallers with low Softmax-predicted probabilities of acceptance, and frequently accepted signallers with high Softmax-predicted probabilities of acceptance.

## 4. Discussion

Decision-makers often need to learn about their signalling environments while simultaneously seeking to maximize their rewards. Here we have highlighted the deep connection between SDT and multi-armed bandits by reframing a classical SDT model as a one-armed contextual bandit (CMAB). In this model, a continuous trait displayed by the signaller indicates (probabilistically) whether the signaller is type A (desirable) or type B (undesirable) and the receiver has the choice of accepting the signaller (thereby gaining uncertain reward) or rejecting it (thereby gaining a certain payoff of 0). Rather than assuming complete information, recasting the SDT model as a CMAB allows receivers to begin with any given prior belief distribution (whether informative or not). This shifts emphasis from asking whether decision-makers use the SDT-predicted rules to asking how decision-makers learn about their signalling environment in a manner that maximizes their payoff [41].

The significant limitations of SDT in requiring complete information have long been recognized [42]. Earlier solutions to the problem have proposed that observers can simply learn the appropriate threshold through a form of reinforcement learning, assuming there is a single cut-off (note that certain ‘improper' types of SDT problem, such as the normal–normal unequal variance model, involve multiple thresholds [2]). In particular, Erev [43] introduced a cut-off reinforcement learning (CRL) model with 10 separate parameters, in which a range (101 in total) of possible discrete cut-offs (thresholds; x*) were hypothesized to have particular propensities to be selected by the receiver (initially normally distributed). For each signaller encountered, a cut-off is then randomly selected by the receiver according to the current propensities, which are then updated through a process of reinforcement based on the nature of the signaller, generalization and forgetting. Unlike our approach, feedback as to the correctness of the decision is gained whatever the response (see also below), which will not always be the case in real-world scenarios. A benefit of the CRL approach is that it is entirely phenomenological and does not seek to estimate the parameters of any underlying generative model (hence it is ‘non-parametric'). However, some drawbacks are: (i) it does not focus on solution efficiency or recognize an exploration–exploitation dilemma at all, (ii) it assumes many fixed parameters and (iii) it imposes additional generalization on the threshold rather than exploiting the properties of the underlying generative distribution. Our parametric approach necessarily assumes that decision-makers can pick out broad patterns in the data they accumulate and thereby estimate the underlying generative model. However, once this assumption is made then the recognition that SDT problems can be framed as CMABs means that researchers can access literature on the range of algorithms that have been proposed to resolve exploration–exploitation problems. This allows investigators to consider CMAB heuristics as candidate solutions to SDT problems when experimental subjects start with incomplete information.

Our approach can also be compared with other approaches to stimulus categorization in the psychology literature. In particular, the Generalized Context Model (GCM) [44] is an exemplar model of categorization in which a novel stimulus is classified into one of several categories based on its similarity to known members of that category. Decision-makers with incomplete information in SDT problems are faced with a similar dilemma, namely, how to treat a signaller with an observed signal *x_o_*, given its history with type A and B signallers, each with particular values of *x*. The GCM seeks to provide a mental representation of the categorization process, but our CMAB model is aimed at understanding how decision-makers might build knowledge in a way that maximizes their long-term payoff. Thus, the GCM model estimates the conditional probability of a signaller with trait *x_o_* being a given type using a summed exponentiated similarity function that can involve both response biases and differential memory strengths. By contrast, our model simply assumes a logistic relationship between the probability of a signaller being type A and *x* and that observers are able to update their prior beliefs as to the logit intercept and slope of this relationship as more experience is gained.

Increasing attention is being given to contextual bandit problems in which side information provides clues as to the payoffs from behaving in a certain way towards the signaller [45,46]. Woodroofe [31] provided the first example of a contextual bandit problem, describing a case in which *N* patients, each with an observable covariate (*x*) such as their age, arrive sequentially for treatment. Each subject may be given either a standard treatment with known benefits given *x*, or a new treatment, whose benefits may depend in some unknown way to *x*. Although seemingly far removed from signal detection problems, the decision-maker in this situation is faced with precisely the same problem of seeking to maximize its cumulative payoff as it learns to identify the best response to a signaller with trait *x*. Clayton [47] likewise presented a contextual bandit model in which the probability of success of a given choice of treatment for a patient depends on both the treatment and a patient covariate (*x*), which took the form of a treatment-specific logistic function of *x*. Here the (logit) intercept and slope were known for one treatment, but were unknown (yet estimable) for another, making it a one-armed contextual bandit of precisely the same form as we have considered in this paper. As in our case, information is only gained from choosing the treatment with an uncertain probability of success, so while it may pay to explore early on, if it is reasonably clear the patient will do better on the standard (known) treatment then this information will be exploited. In some formulations of SDT problems, the observer will always ultimately discover the nature of signaller (A or B) whatever its response to the signaller (treat as A or treat as B). So, independent of how the observer behaves, it will always learn more about the relationship between a signaller's appearance and its probability of being type A. In this case, an observer will continue to update its beliefs (which can be modelled through Bayesian inference) but there is no exploration–exploitation trade-off. With no incentive to go against one's expectations, then the optimal solution to this simpler class of SDT problem will be to be Greedy—i.e. to behave in a way that maximizes the immediate expected reward.

After arguing that CMABs can provide solutions to SDT problems, we asked if and how naive receivers solve SDT problems in practice. By the end of 50 trials, our volunteers gained less on average than a receiver with complete information, but they gained more than a receiver that did not use contextual information and simply accepted signallers at random. This is unsurprising, particularly given that humans have been previously shown to take cues (e.g. continuous [48], binary [49]) into consideration when choosing their course of action in multi-armed bandit tasks. Overall, the Softmax strategy was best able to predict the decisions made by our volunteers, as it has previously for non-contextual bandit problems presented in experiments to humans, including two-armed bandits [50] and four-armed ‘restless' bandits in which reward rates drift over time [51]. Overall, the variability in performance among all learning strategies was lower in higher discrimination conditions because all strategies (with the exception of Random) quickly become effective in accepting type A signallers and rejecting type B signallers.

Somewhat surprisingly, the Midpoint strategy best explained the human choices in three of our four treatments (electronic supplementary material, tables S4–S7). Interestingly, the experimental condition (high base rate, low discriminability) under which the Midpoint strategy was a poor predictor of human choices (electronic supplementary material, table S5) is precisely the same condition it performed poorly in simulations (figure 2). As noted earlier, the optimal thresholds identified in appendix S2 of the electronic supplementary material help explain why the Midpoint strategy is more successful when the signallers are more discriminable. Indeed, when *b* = *c*, *σ* = 25 and the signallers were not readily distinguishable $\left(\mu_{A}=115,\;\mu_{B}=140\right)$ then the true optimal threshold was 21.18 RGB units higher and lower than the midpoint for *ρ =* 0.7 and *ρ =* 0.3, respectively. By contrast, when the signallers were on average more distinguishable $\left(\mu_{A}=90,\;\mu_{B}=165\right)$ then the optimal threshold under the same conditions was only 7.06 units from the midpoint dependent on the base rate (7.06 higher for *ρ* = 0.7 and 7.06 lower for *ρ* = 0.3).

Note that while we can confirm the relative success of some exploration–exploitation strategies, there may be superior strategies that have not been considered in our simulations or fitted in our experiments. In particular, none of the heuristics we have compared take into account the time horizon available to decision-makers. Indeed, if the receiver has any incentive to sample, then rather than accept the first signaller with some intermediate probability, it should always accept this signaller since this information will have the highest value. Supporting this idea, participants in an experimental test of non-CMAB models by Wilson *et al*. [52] were more exploratory the higher the number of trials they were given, suggesting that the rules they were using took into account the future value of information.

Bandit problems, including contextual bandits, have been presented and discussed for several decades, but it is only in the past few years that interest has blossomed—in part due to the increasing popularity of Bayesian methods, but mainly because of their applicability to a range of practical problems, including drug trials [31,53] and the efficient targeting of Internet content [21,54]. Likewise, many biological interactions—ranging from immunological responses to foraging decisions—involve the recognition of stimuli, and SDT provides an intuitive Bayesian framework for identifying the appropriate balance between acceptance and rejection errors [10]. Here we have shown that CMABs offer a principled way to solve many classical SDT problems when decision-makers start with any level of prior information, with SDT representing a special case in which decision-makers adopt the appropriate threshold from the outset.

## Data Availability

Data and code are available from the Dryad Digital Repository: https://doi.org/10.5061/dryad.h44j0zpr3 [55]. The data are provided in electronic supplementary material [56].
